# Diagnosis and management of Neuro-Behçet’s disease: international consensus recommendations

**DOI:** 10.1007/s00415-013-7209-3

**Published:** 2013-12-24

**Authors:** Seema Kalra, Alan Silman, Gulsen Akman-Demir, Saeed Bohlega, Afshin Borhani-Haghighi, Cris S. Constantinescu, Habib Houman, Alfred Mahr, Carlos Salvarani, Petros P. Sfikakis, Aksel Siva, Adnan Al-Araji

**Affiliations:** 1 Neurology Research Department, University Hospital of North Staffordshire, Stoke-on-Trent, England, UK; 2 Central Manchester University Hospital, Manchester, UK; 3 Neurology Department, Bilim University, Istanbul, Turkey; 4 Department of Neurosciences, Saudi Neurology Society, King Faisal Specialist Hospital and Research Centre, Riyadh, Saudi Arabia; 5 Academic Division of Clinical Neurology, Queen’s Medical Centre, University of Nottingham, Nottingham, UK; 6 Health Policy Research Center and Department of Neurology, Shiraz University of Medical Sciences, Shiraz, Iran; 7 Department of Internal Medicine, Faculty of Medicine of Tunis, Hospital La Rabta, University el Manar 2 Tunis, Tunis, Tunisia; 8 Department of Internal Medicine, Hospital Saint-Louis, University Paris 7, René Diderot, Paris, France; 9 Rheumatology Unit, Department of Internal Medicine, Azienda Ospedaliera, IRCCS di Reggio Emilia, Viale Risorgimento n 80, 42123 Reggio Emilia, Italy; 10 First Department of Propedeutic Internal Medicine, Athens University Medical School, Athens, Greece; 11 Neurology Department, Istanbul University, Istanbul, Turkey; 12 Keele University, Keele, Staffordshire UK

**Keywords:** Neuro-Behçet’s disease, Behçet’s disease, Delphi method, Consensus, Diagnosis, Management

## Abstract

Neuro-Behçet’s disease (NBD) is one of the more serious manifestations of Behçet’s disease (BD), which is a relapsing inflammatory multisystem disease with an interesting epidemiology. Though NBD is relatively uncommon, being potentially treatable, neurologists need to consider it in the differential diagnosis of inflammatory, infective, or demyelinating CNS disorders. Evidence-based information on key issues of NBD diagnosis and management is scarce, and planning for such studies is challenging. We therefore initiated this project to develop expert consensus recommendations that might be helpful to neurologists and other clinicians, created through an extensive literature review and wide consultations with an international advisory panel, followed by a Delphi exercise. We agreed on consensus criteria for the diagnosis of NBD with two levels of certainty in addition to recommendations on when to consider NBD in a neurological patient, and on the use of various paraclinical tests. The management recommendations included treatment of the parenchymal NBD and cerebral venous thrombosis, the use of disease modifying therapies, prognostic factors, outcome measures, and headache in BD. Future studies are needed to validate the proposed criteria and provide evidence-based treatments.

## Introduction

Hulusi Behçet, a Turkish dermatologist, described the triad of recurrent oral and genital ulcers with uveitis in 1937 [1]; the disease is commonly referred to as Behçet’s disease (BD) and is recognized as a multisystem inflammatory disorder of unknown aetiology [2]. Interestingly, BD is more prevalent along the ancient Silk Road, including countries in the Far East, the Middle East, and the Mediterranean basin [3]. It has been reported, however, from most countries across the globe [4]. Neuro-BD (NBD) refers to the neurological manifestations of the disease.

The various systemic features of BD and its commonly accepted diagnostic criteria, in addition to the description of the various epidemiological and clinical features of NBD have been described in previous publications [3–5].

In recent years, paraclinical diagnostic tests and an increasing range of immunomodulatory treatments are available for NBD patients. Practice guidelines are needed to improve the diagnostic process, improve quality of care, encourage sensible use of resources, and ensure a balanced consideration of potentially harmful medications. Because NBD is relatively uncommon, studies providing high-quality evidence are very limited. Published studies include mainly personal experiences or single-centre approaches.

Our aim was to reach expert consensus recommendations on the key issues of the diagnosis and management of NBD. As BD is a systemic disease, we felt that wide consultation with an international, multidisciplinary panel was essential to identify the key issues (scope of the recommendations) before reaching consensus through a Delphi exercise amongst a group with a majority of neurologists with special interest in BD.

The Delphi method has been widely used in healthcare and especially in developing clinical practice guidelines where rigorous data are lacking. It includes repeated rounds of communications and voting amongst a panel of experts. The outcome represents the collective opinion of the panel [6, 7].

In this article, we aim to address the key issues in diagnosis and management of NBD. We will present a brief summary of the relevant background literature on each topic of interest, followed by the list of agreed consensus recommendations.

## Methods

### Panel selection

The project was initiated by invitations to a wide range of experts with academic and clinical experience in the field of BD across the globe, who were mostly members of the International Society for Behçet’s Disease. Fifty-two experts from 22 countries accepted the invitation, including a voluntary patients’ group representative. They included 22 neurologists, 11 internists, and 19 other specialists, including 13 rheumatologists, two ophthalmologists, one dermatologist, one immunologist and one paediatrician. The panel was structured into three groups: a project organising committee (POC), a Neuro-Behçet’s advisory group and a Neuro-Behçet’s consensus group. The POC consisted of four members, AA-A, the convenor of the project, SK, the researcher and bibliographer, AS, an academic clinical epidemiologist, and GAD, an experienced neuro-Behçet’s expert. The advisory group consisted of all 52 panel members who participated till the second round of the Delphi process. The consensus group consisted of 12 members (majority neurologists: seven) chosen from the advisory group based on their active contribution in the consensus process, specialty, and publications records. They continued the further steps of the Delphi exercise and are the authors of this paper. The project methodology was discussed, amended, and agreed upon by all participating members before starting the consensus process.

### Search strategy and selection criteria

Literature was searched on Cochrane, Medline, and Embase databases using the key search terms “Behçet *”, “Neuro Behçet *”, and “triple-symptom complex” for entries from 1948 until April 2011. Titles and abstracts of published articles were reviewed. The search was limited to human studies only, published in the English language. The articles addressing diagnosis or treatment were reviewed, which could be case reports, case series, observations, comparison studies, interventional studies, or reviews. These were supplemented by reference lists from the authors’ own collections. Full texts of relevant articles were reviewed and the final reference list was generated on the basis of relevance to the scope of this consensus. The search was updated every 3 months until April 2013. Draft recommendations were generated with their level of evidence; levels I–IV were used to grade the articles [8].

### The Delphi consensus process

We used a four-round Delphi consensus process through email communications. In the first round, we defined the scope of the project and established the need for the diagnostic criteria for NBD and achieved agreement on the disease terminology. The scope consisted of the key issues in the diagnosis and management of NBD that were accepted to be covered in this consensus. This was defined after an extensive literature search, and a list was sent out to the advisory group and amended after the feedback. This round also explored the views on the conflicting disease terminology and voting was done to establish majority’s view and agreement.

**Fig. 1:**
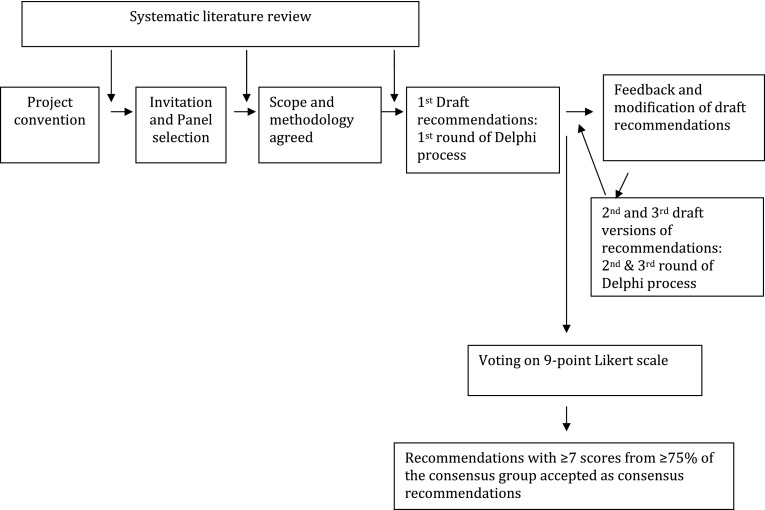
Project methodology

The second and third rounds included sending out draft recommendations derived following the systematic literature review. These were amended following comments from the advisory group and consensus group, respectively. Participants were also sent a background literature review by the researcher/bibliographer. This was an objective summary of the literature review without any opinion or bias from the POC. In the fourth round, the consensus group voted on the third draft version of the recommendations using the 9-point Likert scale with response categories ranging from “strongly disagree” (1) to “strongly agree” (9). Members were asked to express their level of agreement on each recommendation. The final version of each recommendation was accepted as consensus recommendation only if ≥75 % of the consensus group members gave an acceptable agreement score, defined as scores ≥7. Figure 1 depicts our overall project methodology.

## Results

The advisory group agreed on the scope of the project to cover the diagnosis, investigations, and management issues, as shown in Table 1.

**Table 1:** Project scope: key issues addressed in the consensus recommendations

Diagnosis
1. Diagnostic criteria for NBD addressing the certainty of the diagnosis
2. Classification of NBD
3. Differentiating NBD from mimics
Role of investigations
1. Serum inflammatory markers (ESR, CRP and inflammatory cytokines)
2. Neuroimaging
3. CSF parameters
4. IL-6 in serum and CSF
5. Pathergy test
6. HLA B51
7. Neuro-physiological tests—VEP, NCS/EMG
8. Nervous tissue biopsy
Management
1. The role of the followings in the treatment of parenchymal NBD
(a) Steroids
(b) Disease modifying therapies (DMT)
(c) Biological agents
(d) Cyclosporin
2. The role of the following in the treatment of cerebral venous thrombosis
(a) Steroids
(b) Anticoagulation
(c) DMT
3. Prognostic factors
Others
1. Headache in BD
2. Asymptomatic (silent) NBD
3. Outcome measures

**Fig. 2:**
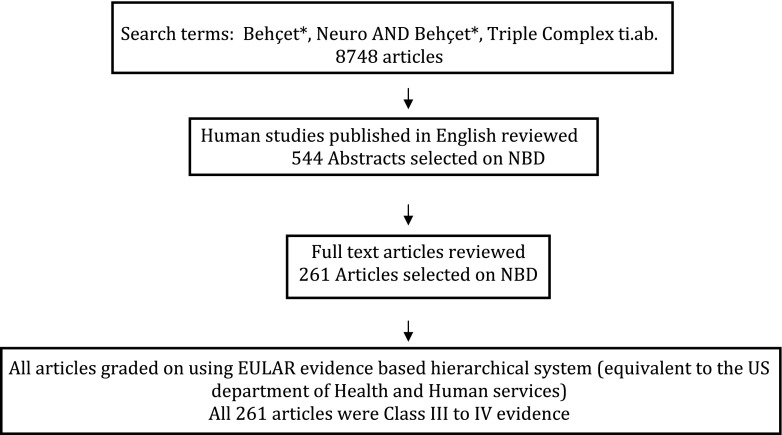
Summary of the systematic literature review

A literature search showed that there were no studies with level I or II in any of the items researched within the scope of this consensus. The best available evidence was in the form of studies within level III or IV (Fig. 2). There are a few randomized, controlled trials investigating treatment options in BD [9, 10] and two meta-analyses, but none on NBD [11, 12]. Figure 2 shows the summary of the literature review and evidence used in the project to draw draft recommendations.

The disease terminology was voted and agreed upon as 'neuro-Behçet’s disease', as two-thirds of the advisory group members preferred to use this term to describe the neurological manifestations of BD (66 % voted for neuro-Behçet’s disease, 22 % for neuro-Behçet syndrome, and 12 % for either/other).

A diagnostic criteria for NBD and 16 recommendations covering important diagnostic and management aspects in NBD were voted through and approved as consensus recommendations. The supporting voting score summary is given in Table 2.

**Table 2:** Voting score summary

Recommendation	Score range	Mode	Median
Recommendation: ICR diagnostic criteria for NBD	7–9	8	8
Recommendation 1: classification of NBD: 1a	5–9	9	9
Onset presentation of NBD subtypes: 1b	7–9	Bimodal 8 and 9	8
Course of subtypes: 1c	7–9	9	9
Recommendation 2: when to consider NBD: 2a	7–9	9	9
How to differentiate: 2b	4–9	Bimodal 8 and 9	8
Recommendation 3: role of inflammatory markers	8–9	9	9
Recommendation 4: role of neuroimaging	5–9	9	9
Recommendation 5: role of CSF: 5a	8–9	9	9
Expected findings in CSF: 5b	6–9	9	9
Recommendation 6: role of IL-6 cytokine	5–9	9	8
Recommendation 7: role of pathergy test	6–9	9	8
Recommendation 8: role of HLA-B51	5–9	9	9
Recommendation 9: role of neurophysiology	6–9	9	9
Recommendation 10: role of nervous tissue biopsy	6–9	9	9
Recommendation 11: management of parenchymal NBD: 11a	8–9	9	9
Role of steroids in parenchymal NBD: 11b	6–9	Bimodal 8 and 9	8
Role of disease modification treatment: 11c	4–9	9	9
Type of disease modification treatment: 11d	4–9	9	8
Role of biological agents: 11e	4–9	9	9
Role of cyclosporin: 11f	4–9	9	9
Recommendation 12: Management of CVT: role of steroids: 12a	7–9	9	9
Role of anticoagulation: 12b	7–9	Bimodal 7 and 9	8
Role of disease modification treatment: 12c	7–9	9	9
Recommendation 13: prognostic factors 13a	6–9	9	9
Poor prognostic factors and treatment: 13b	7–9	9	9
Recommendation 14: headache in BD: 14a	5–9	9	8
Headache at the time of flare ups: 14b	7–9	Bimodal 8 and 9	8
When to investigate: 14c	8–9	9	9
Recommendation 15: asymptomatic (Silent) NBD	7–9	9	9
Recommendation 16: outcome measures 16a	7–9	9	8
Validation of outcome measure: 16b	5–9	9	9

The following section covers the consensus recommendation with a brief summary of the relevant literature review divided into the following subsections:

- Diagnostic criteria for NBD
- Clinical aspects
- Role of investigations in diagnosis of NBD
- Management of NBD
- Miscellaneous

### International consensus recommendation (ICR) diagnostic criteria of NBD

Although BD is a well-defined disease with well-established criteria for its diagnosis [13, 14], the same is not true for NBD. The neurological manifestations of BD have been characterised in many publications [15–19]. Neurological symptoms and signs seen in BD patients are not necessarily due to NBD, and could represent primary neurological disorders like stroke or migraine, or adverse effects of treatment of BD, like secondary infections or malignancy.

There have been previous attempts to produce diagnostic criteria of NBD, but these have not been validated and did not gain general acceptance [12, 18, 20]. Clinically-based diagnostic criteria of other neuro-inflammatory conditions, whether confined to the CNS like in multiple sclerosis [22], or neurological manifestations of a systemic disease like in neurosarcoidosis [23] have inspired us to develop our criteria.

In Table 3, we present practical clinical diagnostic criteria for NBD, approved by consensus. We are not describing a new disease entity, but characterising specific manifestations of a systemic disease. The objective is to help clinicians to diagnose patients with NBD with certainty through identifying the recognised clinical syndromes (presentations) and excluding mimics. Within the criteria, we propose “definite” and “probable” categories to express different degrees of certainty based on the diagnosis of BD and on the details of the neurological presentation. In Table 4, we clarify the terms used in the ICR diagnostic criteria for NBD.

**Table 3:** International consensus recommendation (ICR) criteria for NBD diagnosis

*Definite NBD* meeting all of the following three criteria
1. Satisfy the ISG^a^ criteria for BD
2. Neurological syndrome^b^ (with objective neurological signs) recognised to be caused by BD and supported by relevant and characteristic^c^ abnormalities seen on either or both:
a. Neuroimaging
b. CSF
3. No better explanation for the neurological findings
*Probable NBD* meeting one of the following two criteria in the absence of a better explanation for the neurological findings:
1. Neurological syndrome as in definite NBD, with systemic BD features but not satisfying the ISG criteria
2. A non-characteristic neurological syndrome occurring in the context of ISG criteria-supported BD

^a^ *ISG* International Study Group Criteria 1990 or any other accepted current or future criteria

^b^The recognised syndromes and the ^characteristic findings on investigations are described in Table 4 and in the text

**Table 4:** Clarification of terms used in the ICR diagnostic criteria for NBD

Recognised neurological syndromes
Parenchymal syndrome (one or more of the following presentations at first/subsequent attack(s) or progression)
• Brainstem: symptoms and signs of brainstem involvement including ophthalmoparesis, cranial neuropathy, cerebellar or pyramidal dysfunction.
• Multifocal (diffuse): variable combination of brainstem signs and symptoms, cerebral or spinal cord involvement
• Myelopathy
• Cerebral: symptoms and signs suggestive of cerebral hemispheric involvement including encephalopathy, hemiparesis, hemisensory loss, seizures and dysphasia, and mental changes including cognitive dysfunction and psychosis
• Optic neuropathy
Non-parenchymal syndromes
• Cerebral venous thrombosis
• Intracranial hypertension syndrome (pseudotumour cerebri)
• Acute meningeal syndrome
Characteristic MRI findings in NBD
Parenchymal NBD
Nature of the lesions
• Acute/subacute lesions are hypo-intense to iso-intense on T1-weighted (T1W) images, commonly enhanced with contrast on Gad-T1W images, are hyper-intense on T2W and FLAIR images, hyper-intense on diffusion-weighted images, and show a restricted apparent diffusion coefficient (ADC) on ADC map
• In chronic phase, smaller lesions might be seen, usually non-enhancing, but might resolve completely. There might be evidence of atrophy especially in the brainstem. Nonspecific white matter lesions can be seen
Location: depends on the clinical presentation
• The brainstem is the typical predilection site, lesions usually involving the pons, might extend upwards to involve midbrain, basal ganglia, and the diencephalon
• With cerebral presentation, multiple small, white matter lesions without a clear predisposition for peri-ventricular regions can be seen. Isolated cerebral hemisphere lesions can be seen, which need differentiation from tumour, abscess, and congenital cysts, etc.
• Single or multiple inflammatory lesions of variable length involving the cervical or thoracic cord can be seen, mostly in the presence of brainstem, basal ganglia, or cerebral lesions. Isolated spinal cord lesions are rare
Non-parenchymal NBD
• MR venography or CT venography show evidence of cerebral sinus or vein thrombosis
• Normal appearances are seen in intracranial hypertension syndrome
• Meningeal enhancement is seen in acute meningeal syndrome, especially on Gad-T1W images
Characteristic CSF finding
Inflammatory changes involving one or more of:
• Increased cells
• Increased protein
• High IL-6
Specific conditions to be excluded:
• CNS infections
• CNS neoplasms
• Neurological complications of therapies for BD

### Clinical aspects

#### Classification of NBD

The CNS is the usual site of neurological involvement in NBD. There are two main categories of CNS involvement, parenchymal and non-parenchymal [15–19, 24–26]. The consensus-agreed classification is shown in Table 5, whilst the consensus summary of their clinical details and disease course are highlighted in Table 4.

**Table 5:** Consensus classification of neuro-Behçet’s disease

Central nervous system
Parenchymal
• Multifocal/diffuse
• Brainstem
• Spinal cord
• Cerebral
• Asymptomatic (silent)
• Optic neuropathy
Non-parenchymal
• Cerebral venous thrombosis: intracranial hypertension
• Intracranial aneurysm
• Cervical extracranial aneurysm/dissection
• Acute meningeal syndrome
Peripheral nervous system (relation to BD uncertain)
• Peripheral neuropathy and mononeuritis multiplex
• Myopathy and myositis
Mixed parenchymal and non-parenchymal disease

#### When to consider NBD in a neurological patient

It is important to consider NBD in a neurological patient who has recurrent oral or genital ulcers, uveitis, or other systemic features of BD. NBD has characteristic clinical presentation patterns (Table 4); it is logical to remember BD in the differential diagnosis of these presentations and to ask specifically about the systemic features of BD, even if patients do not volunteer these symptoms.

NBD should be considered in the differential diagnosis of multiple sclerosis (MS) when there are atypical features and especially associated systemic symptoms. Certain neurological features like sensory presentation, optic neuritis, internuclear ophthalmoplegia, limb ataxia, and cerebellar dysarthria are more common in MS, while headaches, motor symptoms, pseudobulbar speech and cognitive-behavioral changes are more common in NBD [27]. The presence of brainstem atrophy in NBD can be used as a powerful discriminator, especially in the absence of atrophy of other brain parts [28]. Spinal cord involvement is less common in NBD [29]. Unmatched cerebrospinal fluid (CSF) oligoclonal bands are present in the majority of MS patients and are uncommon in NBD [30]. CSF shows more cells in parenchymal NBD and neutrophils might predominate, while cells are usually scarce in MS and lymphocytes predominate [31].

Other systemic inflammatory disorders, especially those that might present with uveo-meningitic syndromes including sarcoidosis, systemic lupus erythematosus, and primary Sjögren’s syndrome, are important differentials; occasionally primary CNS lymphoma can present with uveal involvement and a diencephalic lesion. The differentiation requires identification of characteristic clinical patterns in addition to the serological markers and other paraclinical tests [32]; interestingly, the peripheral nervous system is more often involved in non-BD inflammatory diseases.

Acute parenchymal NBD might simulate arterial stroke, which is uncommon in BD patients and when encountered, is more often due to atherosclerosis rather than inflammation (Table 6).

**Table 6:** Recommendations on the diagnosis of NBD

Recommendation 1
(a) There are two main subtypes of NBD: parenchymal, an inflammatory meningo-encephalitic process, and non-parenchymal, which occurs secondary to vascular involvement. These differ by clinical, laboratory, neuro-radiological, pathological, and prognostic characteristics
(b) Parenchymal NBD usually presents with a sub-acute onset of brainstem syndrome with or without other features, cerebral hemispheric or spinal cord syndrome, and features will include pyramidal weakness, behavioural changes, headaches, ophthalmoplegia and sphincter changes. Non-parenchymal NBD commonly presents with headache and visual features secondary to intracranial hypertension, usually due to cerebral venous thrombosis. It can also present as an acute stroke related to arterial thrombosis, dissection, or aneurysm, although this is uncommon
(c) Parenchymal NBD usually follows a relapsing-remitting pattern or a primary/secondary progressive course. Non-parenchymal disease can be monophasic, but recurrences may occur. A mixed parenchymal and non-parenchymal disease presentation can occur
Recommendation 2
(a) We recommend considering NBD in the differential diagnosis of multiple sclerosis, stroke affecting the young, intracranial hypertension, meningo-encephalitis, and myelitis
(b) NBD can be differentiated from its mimics by a combination of characteristic clinical and paraclinical neurological findings in addition to the associated systemic features

### Role of investigations in the diagnosis of NBD

#### Serum inflammatory markers

Although raised ESR and other serum inflammatory markers have been found to be associated with disease activity in BD [10], no definite identifiable pattern has been recognised to be linked with NBD activity. Few studies have reported concurrent appearance or worsening of systemic features and non-specific constitutional symptoms at the neurological presentation, whilst one study reported only modest elevations in inflammatory markers in less than a quarter of NBD patients [18, 19, 33].

#### Neuroimaging

Neuroimaging has a significant role in the diagnosis of NBD; MRI is the gold-standard neuro-imaging modality. MRI abnormalities have been well-described in NBD [34–41]. The consensus characteristics of MRI lesions are listed in Table 4.

MRI is extremely useful in differentiating NBD from its mimics. The brainstem–thalamic–basal ganglia lesions, in the proper clinical context can strongly support the diagnosis of acute/subacute parenchymal NBD, and on occasions can raise this possibility even when the systemic features of BD are scarce [28]. Chronic parenchymal NBD lesions are iso-intense, smaller, and at times difficult to differentiate from lesions seen in multiple sclerosis.

In general, multiple sclerosis lesions are predominantly periventricular, with infrequent involvement of the basal ganglia, internal capsule, and the peripheral part of the pons, whilst chronic parenchymal NBD lesions are predominantly subcortical. Brainstem atrophy in association with subcortical lesions points toward NBD [28]. In neuro-Lupus, though sub-cortical white matter lesions are seen, basal ganglia or brainstem involvement is uncommon [31].

#### CSF

Cerebrospinal fluid constituents are altered in around 70–80 % of patients with parenchymal NBD [14–17]. CSF protein is modestly raised in most cases, and oligoclonal bands are usually absent [19, 30, 33]. The CSF cell count is raised in 60–80 % of parenchymal NBD cases (range 0–400 × 10 cells/L) and there could be CSF neutrophilia, lymphocytosis, or mixed cellularity [15, 16, 19]. CSF glucose is usually normal in NBD and low levels point toward CNS infections [15].

Patients with CVT or intracranial hypertension without CVT (pseudotumour cerebri) have normal CSF constituents, but usually high CSF opening pressure.

#### IL-6 cytokine

Serum IL-6 levels have been reported to correlate with BD disease activity, although this finding has not been consistently reproduced [42, 43].

Raised CSF levels of IL-6 have been seen in patients with acute parenchymal NBD [43–49]. A smaller rise in IL-6 levels has also been reported in a proportion of progressive parenchymal NBD [21, 43]. Raised CSF IL-6 levels are usually associated with raised CSF cell count and protein, and these three parameters have been associated with disease activity and outcome over 3 years. Occasionally elevated CSF IL-6 levels were reported in the presence of normal CSF cells and protein [21, 43].

Japanese studies have shown reductions in CSF IL-6 levels in response to various treatments, but it is difficult to draw a clear conclusion, as these studies involved small numbers of patients [21, 44, 46, 47].

Collectively, these data indicate that IL-6 is not a reliable biomarker of NBD or BD, and the absence of IL-6 should not be viewed as the absence of disease activity. In addition, little is known about the normal expected levels of IL-6 in the CSF.

#### Pathergy test

The pathergy test is one of the major criteria in BD diagnosis [14]. Pathergy reaction is a non-specific hyperreactivity of the skin to trauma, such as a needle prick. A positive test is defined as a papule or pustule that typically appears 24–48 h after an intradermal injection of the skin with a 20-gauge needle. Pathergy positivity is highly suggestive but not pathognomonic of BD.

False positive tests can be seen in pyoderma gangrenosum, Sweet syndrome, inflammatory bowel diseases, familial Mediterranean fever, acute myeloid leukemia, and interferon alpha treatment [48, 49]. Pathergy test sensitivity has significant geographical variation. While 60–70 % of Turkish and Japanese BD patients have a positive test, it is uncommon in Northern European and North American BD patients [49, 50].

#### HLA-B51

BD is associated with the major histocompatibility complex HLA-B5 allele and, more specifically, with HLA-B51 [11].

HLA-B51/B5 prevalence varies across the globe, being higher in Asian, Middle Eastern, and Southern European populations, and lower in Northern Europe and North America [11]. Overall, the HLA B5 genotype is seen in 40–65 % of patients diagnosed with BD, and in 10–20 % of healthy individuals of ethnically-matched control populations [11]. The relatively modest sensitivity and specificity of HLA-B51/B5 imply that HLA class 1 genotyping has only a limited value as diagnostic test. In addition, the prevalence of HLA-B51/B5 among subjects with NBD is not dissimilar to that found in patients with BD without neurological involvement [12]. Therefore, testing for HLA-B51/B5 does not appear to provide a substantial aid for the diagnosis of NBD. Conflicting findings have been reported as to whether or not HLA-B51/B5 status may predict a more severe BD course [51, 52].

#### Neurophysiology tests

Neurophysiological testing may be useful if peripheral nervous system involvement or optic nerve involvement is suspected. Although neurophysiology tests can detect central or peripheral nervous system involvement, MRI remains the gold standard for CNS involvement. Occasionally, EEG can be useful in the differential diagnosis from acute viral encephalitis [5]. Visual-evoked potential (VEP) can detect optic nerve involvement, however, frequent uveal involvement in BD might hamper the usefulness of VEPs [53].

A number of studies have reported abnormal findings on neurophysiological testing in the absence of clinical signs or symptoms [54–57]. These include asymptomatic abnormalities on nerve conduction studies, electromyography, and motor-, sensory- or brainstem-evoked potentials. The significance of these abnormalities is uncertain, and caution should be exercised before these are taken as evidence of central and/or peripheral nervous system involvement. The diagnosis of NBD should not be made solely on the basis of these abnormalities.

#### Nervous tissue biopsy

The pathologic findings of CNS involvement in BD are not pathognomonic, but are well-described in the literature [58–60]. The basic pathology in the acute/subacute parenchymal presentation is a perivasculitis characterised by perivascular infiltration with lymphocytes, neutrophils, and rarely, eosinophils with or without signs of necrosis. In later stages, inflammatory infiltration is less prominent, and axonal loss and gliosis predominate [58–60].

The clinical presentation, neuroimaging, and CSF findings are usually sufficient to secure a diagnosis without the need for a tissue diagnosis. Tumour-like presentation, though uncommon, has been reported in the literature [61, 62]. Careful history taking in such patients commonly reveals the systemic symptoms of BD, and help in the early diagnosis. Occasionally, a tissue diagnosis is needed after all other diagnostic avenues have been used (Table 7).

**Table 7:** Recommendations on the role of investigations in diagnosis of NBD

Recommendation 3
ESR, CRP, and inflammatory cytokines are non-specific markers of inflammation; these might be elevated at the neurological presentation, but are of limited value in the differential diagnosis of NBD
Recommendation 4
We recommend considering MRI study including contrast and MRV in suspected NBD. This commonly demonstrates characteristic features especially in acute/sub-acute parenchymal involvement and can confirm CVT. The distinct MRI findings are helpful in the differentiation from the other CNS inflammatory disorders
Recommendation 5
(a) We recommend CSF examination in suspected NBD, as it has a supportive role in the diagnosis, in addition to looking for mimics and especially CNS infections
(b) Parenchymal NBD is usually associated with CSF pleocytosis (either neutrophilic or lymphocytic, but rarely as florid as seen in bacterial meningitis), and/or raised protein. Oligoclonal bands are frequently absent. A completely normal CSF does not exclude parenchymal NBD. Non-parenchymal NBD is associated with elevated CSF pressure only. The role of CSF abnormalities in prognosis and monitoring of the disease needs further research
Recommendation 6
Raised CSF IL-6 is an indicator of ongoing disease activity in NBD, usually in association with raised CSF constituents. While we recommend considering CSF IL-6 for disease monitoring, especially in the absence of other raised inflammatory CSF constituents, its use in monitoring therapeutic response needs further research
Recommendation 7
The pathergy test is simple and has a well-established role in BD diagnosis. We recommend pathergy testing in suspected NBD, since a positive result, especially with other systemic BD features, would contribute significantly toward NBD diagnosis. A negative test, however, will not exclude NBD
Recommendation 8
BD is associated with the HLA-B5 allele and, more specifically, with HLA-B51. It is not clear if HLA-B51/B5 testing has a role in the diagnosis or prognosis of BD or NBD
Recommendation 9
Neurophysiologic tests are not routinely recommended for NBD. These may be useful if peripheral nervous system or optic nerve involvement is suspected. Asymptomatic neurophysiological findings are of doubtful clinical significance. The diagnosis of NBD should be avoided when solely based on asymptomatic neurophysiological findings
Recommendation 10
Nervous tissue biopsy can occasionally be useful in the diagnosis of NBD. It is usually not recommended as a part of the diagnostic process. As it is an invasive procedure, we recommend considering it when all other diagnostic avenues have been exhausted, especially for tumour-like presentation

### Management of NBD

#### Treatment of parenchymal NBD

There have been no controlled or comparative trials on the treatment of any aspect of NBD [63]. Most neurologists with experience in the management of NBD treat a relapse or acute presentation with daily 1 g IV methylprednisolone infusions, followed by a slowly tapering course of oral steroids in parallel to treatment given for other CNS neuroinflammatory relapses like neuro-Lupus and neurosarcoidosis [22, 64]. It is important to avoid an abrupt cessation of therapy to avoid early relapse. The dose and duration of the initial IV treatment and the subsequent oral therapy vary between different centres [6, 63].

Retrospective studies have shown that two-thirds of patients with brainstem lesions or cerebral lesions make good recovery in response to the courses of steroids, but the other third have recurrence of relapses or progressive course [15–18].

The timing of the start of disease modifying therapies (DMT) is not always straightforward. The rationale is to help in controlling the inflammatory process, to prevent or reduce the frequency of further neurological relapses, to reduce steroid exposure, and possibly to control the other systemic features of this multisystem disease.

Azathioprine was reported to prevent inflammation of the second eye after the first ocular episode in BD [9, 10]. Because of its relatively predictable and low side effects profile, azathioprine is commonly used as a first DMT in many centres for the serious manifestations of BD, including NBD. There are other publications reporting success with alternative DMTs for NBD, including mycophenolate mofetil [65], methotrexate [66, 67], chlorambucil [68], and cyclophosphamide [69].

Infliximab was reported to be effective in treating refractory ocular and NBD, and in achieving favourable outcome [70–72], with continued benefit in follow-up studies over 1- and 4-year periods [73, 74]. Adalimumab has been reported as an effective alternative to infliximab [75, 76]. There are case reports supporting the use of etanercept [77], Tocilizumab [78] and interferon alpha [79, 80]. To date, experience with infliximab is considerably larger compared to other anti-TNF agents [81].

Cyclosporin is effective in the treatment of ocular BD, but has been linked with higher risk of NBD development [10, 82–85].

#### Treatment of cerebral venous thrombosis (CVT)

CVT is a characteristic pattern of NBD presentation. Anticoagulation is the standard treatment of systemic venous thrombosis and CVT of any aetiology [86]. On the other hand, the usage of anticoagulants in CVT due to NBD is controversial and a matter of debate between experts [87]. There are no high-quality data to support the contradictory opinions. The rationale for the difference is that the advocates for avoiding anticoagulants believe that CVT in BD is due to an inflammatory process and that the thrombus formed is tightly adherent to the vessel wall [88], which necessitate the use of anti-inflammatory agents only. Moreover, the possibility of bleeding after the rupture of a coexisting aneurysm anywhere in the body may have detrimental consequences [10, 89]. The supporters of anticoagulants argue that they will consider the use of anti-inflammatory medications to combat the presumed inflammatory aetiology, but they prefer to use anticoagulants, after excluding systemic aneurysms, at least to reduce the risk of further extension of the clot in the cerebral venous system. Our consensus group was split almost equally on both sides of the argument.

The duration of anticoagulant use varies, but is usually around 3–6 months in uncomplicated cases [90]. The duration will be probably for life if clear evidence for an underlying pro-thrombotic status is found.

#### Prognostic factors

Two major retrospective case series on NBD [16, 17] and another study with some prospective data [18] have consistently reported brainstem or spinal cord presentation, frequent relapses, early disease progression, and high CSF pleocytosis as poor prognostic factors. Disability and dependent status at initial presentation, a primary or secondary progressive course, relapse during steroid dose tapering, fever, meningeal signs, and bladder involvement showed a possible association with poor outcome, as defined by poor survival and dependant status [18]. Factors such as gender, presence of other systemic manifestations of BD, and age at onset did not have any influence [18].

Although there are no data from RCTs, with the available evidence from personal experiences, the early use of DMT might be considered where one or more poor prognostic factors are encountered. Other relevant factors also need to be considered in this decision (Table 8).

**Table 8:** Recommendations on the management of NBD

Recommendation 11
(a) There is no level I evidence on the treatment options of NBD. The following recommendations are mainly based on observational data
(b) For acute/sub-acute parenchymal NBD attack, a course of corticosteroids is recommended, preferably IV methyl prednisolone for 3–10 days followed by a maintenance oral corticosteroid for a few months (up to 6 months)
(c) We recommend considering a disease modifying therapy (DMT) after a significant parenchymal relapse depending on severity, response to steroid, previous neurological relapses, disease course, and other associated systemic BD features
(d) Azathioprine is recommended as a first-line DMT; alternatives include mycophenolate mofetil, methotrexate, and cyclophosphamide
(e) We recommend considering a biological agent, including TNF-alpha-blockers (infliximab, adalimumab, etanercept) or interferon alpha, when first=line therapies are ineffective or intolerable and when the disease is relapsing or showing aggressive neurological or systemic features
(f) We recommend caution in using cyclosporin in BD patients because of the potential association with neurological complications. It should be avoided in patients with a history of NBD and the medication should be stopped when BD patients develop neurological features suggestive of parenchymal CNS involvement
Recommendation 12
(a) For CVT in BD, we recommend the use of corticosteroid for a limited period for the acute/sub-acute presentation
(b) There is no convincing evidence to use or withhold the use of anticoagulants, which is a standard treatment of CVT of any aetiology. If anticoagulation is to be started, caution should be taken to rule-out a systemic aneurysm
(c) We recommend considering a DMT, especially if there is a previous history of CVT, active systemic disease, or a history of associated parenchymal NBD
Recommendation 13
(a) Poor prognostic features of NBD include brainstem or myelopathy presentation, frequent relapses, early disease progression, and presence of CSF pleocytosis in parenchymal NBD
(b) We recommend early consideration of a disease modifying treatment when one or more poor prognostic features are encountered

### Miscellaneous

#### Headache

The literature on headache in BD was reviewed in the previous Lancet Neurology paper [5], which summarised all relevant, major, published case series.

Headache is the most common neurological symptom in patients with BD. Most of these headaches are due to primary headache disorders, commonly migraine and tension-type headaches [91]. Aykutlu et al. [92] have shown that the characteristics of the primary headaches in BD patients were not different from those seen in the general population presented to headache clinics. Only about 10 % of BD patients with headaches are due to direct neurological involvement [5]. These are usually seen in association with other neurological features. Headaches have been reported at the time of flare up of systemic BD features in the absence of CNS involvement [91–93]. Recognition of the different types of headaches in BD patients might reduce the unnecessary expenditure and risk of specialist investigations.

#### Asymptomatic (silent) NBD

Asymptomatic abnormalities on neurological examination, neuroimaging, neuropsychology or neurophysiology testing, which are referred to as asymptomatic NBD, have been reported [5, 26]. The significance of these findings is not clear. In a retrospective comparative study, four of 22 BD patients with no specific neurological symptoms but abnormal findings on MRI or neuropsychology testing developed NBD attacks after a mean follow-up of about 13 years [57]. The outcome was milder compared to a group of 55 symptomatic NBD patients.

#### Outcome measures

There is no validated scale for measuring disability in NBD. The following three potential scales could be considered. Two of these require a neurologist’s input with special training.

The modified Rankin scale is internationally accepted and well-validated for the measurement of disability in cerebro-vascular diseases [94–97]. This scale measures overall functional ability and does not focus on individual functional system/domain like cognition. It is easy to perform and can be used without special training. The scores range from 0 to 6.

Expanded disability status scale (EDSS) is validated and is the most widely utilized assessment tool in MS [98]. Some studies have used EDSS to measure neurological disability in NBD, but it has not been validated. The complexity and the technical skills required for its use make it difficult to be adopted by non-neurologists in routine practice.

Neuro-Behçet’s disability score (NBDS) has been proposed for parenchymal-NBD patients to quantify disabilities [99]. This comprises scores for motor and cognitive status. NBDS is the arithmetic sum of both scores and ranges from 0 to 8, with 8 being death due to NBD. Although it appears to be more thorough than the Rankin scale, in the absence of validation this more complex score would be difficult to interpret and extrapolate (Table 9).

**Table 9:** Miscellaneous

Recommendation 14
(a) Headaches in BD patients are commonly due to primary headache disorders like migraine and tension-type headaches
(b) Although headache is one of the most common presenting symptoms of NBD, headache might recur predominately around the time of flare-ups of systemic BD symptoms without evidence of CNS involvement. Recognition of this type of headache might reduce unnecessary and repeated investigations for the possibility of CNS involvement. This type of headache needs further research and clarification
(c) We recommend that BD patients with headaches be considered for further evaluation and investigations when their headaches are progressive, refractory or persistent, severe or incapacitating, if it is the first and worst headache, if there is a change in character, and especially if there are associated neurological symptoms and signs
Recommendation 15
Asymptomatic NBD refers to subtle asymptomatic findings on neurological examination and/or neurological investigations. Its significance is not clear. Current evidence does not support the use of preventive immunosuppressive treatment, and further evidence is required
Recommendation 16
(a) We recommend the use of the modified Rankin scale to measure disability in NBD, as it is simple, can provide a good overall assessment, and can be easily used in clinical practice
(b) We recommend future research to validate this scale in NBD

## Comments and conclusions

We present recommendations for the key issues in the diagnosis and management of NBD, which are intended for the use of practicing clinicians.

Recommendations are a way to support effective clinical practice. While uncertainties, especially in dealing with uncommon conditions, are likely to persist, recommendations can aid clinicians in determining the best options for a particular patient.

The strengths of our consensus recommendations include an extensive literature review and the use of the best available evidence, wide-scale consultations with international experts, involvement of a patient group representative, emphasis on issues that are of particular interest to clinical practice, and setting a high level for accepting a consensus recommendation.

The limitations include lack of high-grade, evidence-based data on all of the issues covered by this consensus, and reliance on level III and IV evidences and experts’ opinions to reach the consensus. There are inherent limitations to the consensus process and the Delphi method.

The proposed diagnostic criteria for NBD include two levels of certainty, but with strict requirements including objective neurological signs to reduce false positive diagnosis and improve accuracy. It only uses investigations, which have well-established supportive roles in the diagnosis. Its use is facilitated by the clear explanations for the terms used. We would like to emphasise that it is a clinical and not pathological criteria. It would require some neurological expertise to characterise the neurological syndromes. It might not help in the diagnosis of difficult and controversial neurological presentations of NBD and caution needs to be practiced, as its accuracy, sensitivity and specificity are unknown while validation is needed.

The use of anticoagulants in Behçet’s venous thrombosis and CVT is an important topic that needs priority in future research. International cooperation is needed to establish future studies on the best treatment options for NBD patients. These recommendations need to be updated in the future, pending further evidence.

## Advisory group members

In addition to the authors of this paper, the following subjects were members of the advisory group: Canada—Prof Simon Carette (Medicine); Egypt—Prof Samir Helmy Assaad-Khalil (Internal Medicine), Prof Sahar N Saleem (Neuro-radiology); France—Prof Loic Guillevin (Internal Medicine), Prof Isabelle Kone-paut (Paediatrics); Germany—Dr. Andreas Altenburg (Dermatology), Dr. Peter Berlit (Neurology), Dr. Thomas Stache (Neurology); Greece—Prof Panagiota Boura (Internal Medicine and Clinical Immunology); Israel—Prof Eldad Ben-Chetrit (Rheumatology); Italy—Dr. Loredana La Mantia (Neurology, Neuropathology), Dr. Ignazio Olivieri (Rheumatology); Japan—Prof Shunsei Hirohata (Rheumatology and Internal Medicine); Jordan—Dr. Wafa Madanat (Rheumatology); Libya—Prof Khaled Elmuntaser (Internal Medicine and Rheumatology); Netherlands—Dr. Jan VanLaar (Internist, Immunologist and Oncologist); Qatar—Dr. Thurayya Arayssi (Rheumatology); Republic of Ireland—Dr. Mary Keogan (Immunology); Spain—Dr. Norberto Ortego-Centeno (Internal Medicine), Dr. Alex Olivé (Rheumatology), Dr. Sergio Martínez-Yélamos (Neurology), Dr. Cristina Ramo-Tello (Neurology), Dr. Roser Solans (Internal Medicine and Autoimmune Diseases); Switzerland—Dr. Oliver Findling (Neurology), Prof Marcel Arnold (Neurology); Turkey—Prof Ayse Altintas (Neurology), Dr. Murat Kürtüncü (Neurology), Prof Salih Pay (Rheumatology and Internal Medicine); UAE—Dr. Sarmed Al Fahad (Neurology); United Kingdom—Dr. John Bamford (Neurology), Prof Dorian O Haskard (Rheumatology), Dr. Desmond Kidd (Neurology), Mrs. Janet Mather (Patient Representative), Prof Philip I. Murray (Ophthalmology), Prof Neil Scolding (Neurology), Prof Miles Stanford (Ophthalmology); United States—Dr. Kenneth T. Calamia (Rheumatology), Dr. Luis R. Espinoza (Rheumatology), Dr. Nadera J. Sweiss (Medicine), Dr. Nagagopal Venna (Neurology).
